# Molecular complete remission following combination treatment of daratumumab and venetoclax in an adolescent with relapsed mixed phenotype acute leukemia

**DOI:** 10.1007/s00277-023-05083-y

**Published:** 2023-01-18

**Authors:** Martin Stanulla, Denis M. Schewe, Beat Bornhauser, Jean-Pierre Bourquin, Cornelia Eckert, Wolfgang Eberl, Saskia Wolf, Julian Wolf, Fotini Vogiatzi, Anke K. Bergmann, Gunnar Cario, Rita Beier, Martin Sauer, Christian P. Kratz, Britta Maecker-Kolhoff

**Affiliations:** 1 Department of Pediatric Hematology and Oncology, Hannover Medical School, Carl-Neuberg-Str. 1, D-30625, Hannover, Germany; 2 Department of Pediatrics, Otto-von-Guericke University Magdeburg, Magdeburg, Germany; 3 Division of Pediatric Oncology, and Children Research Center, University Children’s Hospital, Zurich, Switzerland; 4 Charité, University Hospital Berlin, Pediatric Hematology/Oncology, Berlin, Germany; 5 Center for Child and Adolescent Medicine, Department of Hematology and Oncology, Städtisches Klinikum Braunschweig gGmbH, Braunschweig, Germany; 6 Pediatric Hematology/Oncology, University Hospital Schleswig-Holstein, Campus Kiel, Kiel, Germany; 7 Institute of Human Genetics, Hannover Medical School, Hannover, Germany

**Keywords:** Acute lymphoblastic leukemia, Mixed phenotype acute leukemia, Daratumumab, Venetoclax

**Fig. 1:**
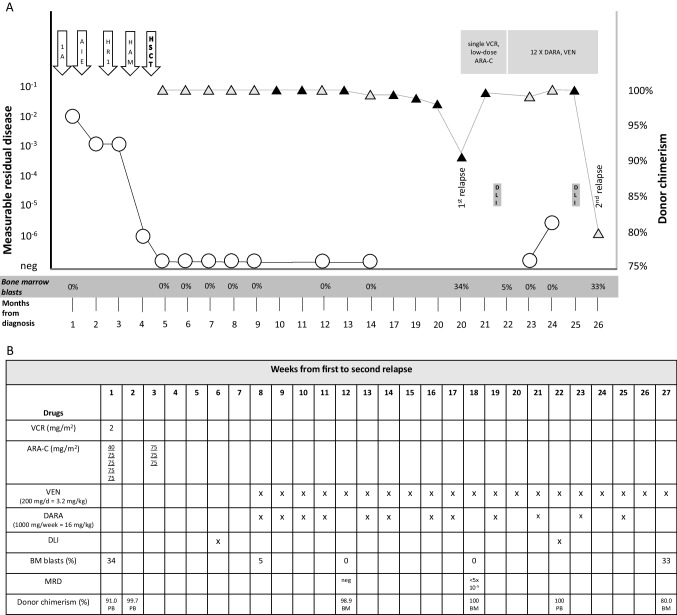
Treatment course of a patient with with *KMT2A::MLLT4*-positive mixed phenotype acute leukemia (T cell ALL/AML). **A** Measurable residual disease (MRD, circles) and donor chimerism measurements (bone marrow: grey triangles, peripheral blood: black triangles) are depicted from initial diagnosis to diagnosis of second relapse. Treatments are indicated in the upper part: 1A, ALL-BFM induction protocol 1A; AIE, cytarabine, idarubicin, etoposide; HR1, ALL-BFM high-risk block 1 with dexamethasone, vincristine, methotrexate, cyclophosphamide, cytarabine, L-asparaginase, intrathecal triple therapy; HAM, high-dose cytarabine, mitoxantrone; HSCT, allogeneic hematopoietic stem cell transplantation; VCR, vincristine; ARA-C, cytarabine. Blast counts below the time axis relate to morphological bone marrow examinations. Time axis not regular. **B** Detailed description of combined treatment with daratumumab (DARA) and venetoclax (VEN). DLI, donor lymphocyte infusion; BM, bone marrow

Dear Editor:

Here, we report on a twelve-year-old boy diagnosed with mixed phenotype acute leukemia (T cell ALL/acute myeloid leukemia, AML) bearing a *KMT2A::MLLT4* fusion in June 2018. First-line treatment was initiated according to the AIEOP-BFM ALL 2009 registry protocol and demonstrated a poor response to the prednisone prephase and high leukemic cell loads by measurable residual disease (MRD) during and after induction therapy (Fig. 1A) [1, 2]. After subsequent application of an AML-type treatment (AIE regimen: cytarabine, idarubicin, and etoposide), an ALL-BFM high-risk block (dexamethasone, vincristine, methotrexate, cyclophosphamide, cytarabine, L-asparaginase, and intrathecal triple therapy), and high-dose cytarabine/mitoxantrone (HAM), the MRD level finally fell to a positive result in the non-quantifiable range (10^-6^, Fig. 1A) [2, 3]. The patient proceeded to hematopoietic stem cell transplantation (HSCT) from an unrelated 11/12 HLA-identical donor (10/2018; conditioning regimen: fludarabine, treosulfan, thiotepa, and anti-thymocyte globulin) [4]. Post HSCT, multiple complications occurred including invasive pulmonary aspergillosis, macrophage activation syndrome, and biopsy-proven thrombotic microangiopathy of the kidneys and lungs—the latter leading to chronic renal and pulmonary insufficiency with pulmonary artery hypertension and chronic oxygen demand. Eculizumab was administered until 04/2020 [5] when the patient experienced an isolated bone marrow relapse. Leukemic blasts carried CD38 as the sole drug target in flow cytometric analyses (positivity 97%). Due to the physical condition and limited prognosis in this situation and after discussion with the family, a palliative treatment approach aiming at low toxicity with low-dose cytarabine and one dose of vincristine was initiated, but only poorly tolerated (hematologic toxicity). *Ex vivo* drug-profiling of leukemic blasts did not point towards specific highly effective compounds, but suggested intermediate sensitivity to the BH-3 mimetic venetoclax (data not shown) [6]. In the sixth week after relapse diagnosis, donor lymphocytes were infused (DLI; 1 × 10^7^/kg body weight). As prolonged hematologic toxicity, nausea, and vomiting triggered by the initial treatment were not compatible with an acceptable quality of life, but the patient and his family strongly wished to continue anti-leukemic treatment, we started a combination treatment with daratumumab infusions (single dose 1000 mg; 16 mg/kg body weight) and oral low-dose venetoclax (single dose 200 mg; 3.2.mg/kg body weight) as an individual patient treatment after information and consent by the patient and guardians [7–9]. When starting this regimen (17-06-2020), the patient still had 5% blasts in the bone marrow by morphology (week 8; Fig. 1B). After four weekly daratumumab applications and daily venetoclax, the bone marrow was morphologically cleared of leukemic blasts and became MRD-negative (Fig. 1A and B). After the eighth daratumumab infusion and continuation of venetoclax, the patient converted to 100% donor chimerism. Since then, the patient received daratumumab on a bi-weekly schedule under continuous exposure to low-dose venetoclax and remained in good clinical condition. One week after the tenth daratumumab infusion, a second DLI was applied. Two weeks after the twelfth daratumumab application, the patient presented for a routine bone marrow evaluation which demonstrated 33% leukemic blasts (CD38-negative) and a donor chimerism of 80%. The treatment with daratumumab and venetoclax was discontinued. During the 20-week treatment period with daratumumab and venetoclax, no serious adverse reactions were noticed. The patient’s quality of life at this stage was excellent. In the further course, the patient received low-dose cytarabine, two additional DLI doses, and mistletoe therapy. The leukemia continuously progressed, and the patient died twelve weeks after diagnosis of the second relapse of cardiac arrest due to respiratory failure.

Providing mechanistic support for our observation, a recent study demonstrated that the addition of venetoclax potently enhances the efficacy of therapeutic antibodies including daratumumab in CD38-positive malignancies, because of an increase in antibody-dependent cellular phagocytosis by macrophages [10]. The unexpectedly good disease control without a significant chemotherapy backbone and the beneficial toxicity profile observed in our patient provide additional clinical support for the potential efficacy of venetoclax/daratumumab and suggests that this combination should be further evaluated in ALL. This is to our knowledge also the first report of CD38 antigen-escape after daratumumab therapy in acute leukemia.
